# Cognitive impairment in long-COVID and its association with persistent dysregulation in inflammatory markers

**DOI:** 10.3389/fimmu.2023.1174020

**Published:** 2023-05-23

**Authors:** Rodolfo Furlan Damiano, Cristiana Castanho de Almeida Rocca, Antonio de Pádua Serafim, Jennifer M. Loftis, Leda Leme Talib, Pedro Mário Pan, Edecio Cunha-Neto, Jorge Kalil, Gabriela Salim de Castro, Marilia Seelaender, Bruno F. Guedes, Suely K. Nagahashi Marie, Heraldo Possolo de Souza, Ricardo Nitrini, Euripedes Constantino Miguel, Geraldo Busatto, Orestes V. Forlenza

**Affiliations:** ^1^ Departamento e Instituto de Psiquiatria, Hospital das Clínicas da Faculdade de Medicina da Universidade de São Paulo (HCFMUSP), São Paulo, SP, Brazil; ^2^ Instituto de Psicologia, Universidade de São Paulo, São Paulo, SP, Brazil; ^3^ Research & Development Service, VA Portland Health Care System, Portland, OR, United States; ^4^ Departments of Psychiatry and Behavioral Neuroscience, Oregon Health and Science University, Portland, OR, United States; ^5^ Departamento de Psiquiatria, Universidade Federal de São Paulo, São Paulo, SP, Brazil; ^6^ Departamento de Cínica Médica, Universidade de São Paulo FMUSP, São Paulo, SP, Brazil; ^7^ Institute for Investigation in Immunology/National Institutes for Science and Technology (iii/INCT), São Paulo, Brazil; ^8^ Cancer Metabolism Research Group, Department of Surgery and LIM 26, Hospital das Clínicas, University of São Paulo, São Paulo, SP, Brazil; ^9^ Departamento de Neurologia, Universidade de São Paulo FMUSP, São Paulo, Brazil; ^10^ Departamento de Emergências Médicas, Universidade de São Paulo FMUSP, São Paulo, SP, Brazil

**Keywords:** COVID-19, SARS- CoV-2, cognition, inflammation, cohort study (or longitudinal study)

## Abstract

**Objective:**

To analyze the potential impact of sociodemographic, clinical and biological factors on the long-term cognitive outcome of patients who survived moderate and severe forms of COVID-19.

**Methods:**

We assessed 710 adult participants (Mean age = 55 ± 14; 48.3% were female) 6 to 11 months after hospital discharge with a complete cognitive battery, as well as a psychiatric, clinical and laboratory evaluation. A large set of inferential statistical methods was used to predict potential variables associated with any long-term cognitive impairment, with a focus on a panel of 28 cytokines and other blood inflammatory and disease severity markers.

**Results:**

Concerning the subjective assessment of cognitive performance, 36.1% reported a slightly poorer overall cognitive performance, and 14.6% reported being severely impacted, compared to their pre-COVID-19 status. Multivariate analysis found sex, age, ethnicity, education, comorbidity, frailty and physical activity associated with general cognition. A bivariate analysis found that G-CSF, IFN-alfa2, IL13, IL15, IL1.RA, EL1.alfa, IL45, IL5, IL6, IL7, TNF-Beta, VEGF, Follow-up C-Reactive Protein, and Follow-up D-Dimer were significantly (p<.05) associated with general cognition. However, a LASSO regression that included all follow-up variables, inflammatory markers and cytokines did not support these findings.

**Conclusion:**

Though we identified several sociodemographic characteristics that might protect against cognitive impairment following SARS-CoV-2 infection, our data do not support a prominent role for clinical status (both during acute and long-stage of COVID-19) or inflammatory background (also during acute and long-stage of COVID-19) to explain the cognitive deficits that can follow COVID-19 infection.

## Introduction

Our continued experience with COVID-19 has led to the identification of numerous extrapulmonary consequences of SARS-CoV-2 infection (1). Of particular relevance to the present report are the psychiatric and cognitive symptoms associated with this infection. Such symptoms were initially identified in large epidemiological studies (2, 3). However, epidemiological studies do not address whether these symptoms are related to the specific pathological consequences of the infection itself, or the social situations of the individuals who contract the virus. More recent cohort COVID-19 studies have demonstrated a significant increase in psychiatric and cognitive symptoms in individuals previously infected by COVID-19, irrespective of the severity of the acute disease; this is true in both mild (4) or more severe forms (5) of the disease.

Numerous studies have documented the effect of SARS-CoV-2 infection on cognition (6, 7). Preclinical studies in mice have demonstrated cognitive deficits in mice after injection of SARS-CoV-2 spike protein directly into the hippocampus (8). In humans, higher cognitive impairment has been reported in post-COVID-19 survivors. Indeed, these post-acute sequelae syndrome (PASC) has been termed long-COVID-19; defined as displaying COVID-19 related symptoms that persist after 3 months following initial infection and lasting for more than 2 weeks. Critically, this impairment is not related to any pre-existing clinical or emotional disturbances (9). Further, a recent report has shown a potential link between a deficit in cognitive performance and chemosensory impairment in long-COVID-19 patients (10, 11). Finally, population studies assessing electronic medical records of over a million individuals confirm the importance of cognitive impairment among post-COVID19 patients (12) and point to an urgent need for gathering researchers and public leaders to better understand and more effectively face the long-COVID-Challenge (13).

One of the most puzzling aspects associated with this research area is the role of inflammation during the acute and post-acute COVID-19 phase and its impact on cognition (14). In general, studies across numerous brain-based disorders have demonstrated a convincing relationship between inflammatory markers and cognitive decline (15), especially in patients with Alzheimer’s disease (16) and chronic viral infections (e.g., HIV, HCV) (17–21). However, studies assessing the relationship between COVID-19 and inflammation remain preliminary in nature. Preclinical data in hamsters are consistent with post-mortem brain data from COVID-19 victims; that is, neural inflammation is one of the core symptoms of SARS-CoV-2 exposure (22). This is relevant because a recent human COVID-19 cohort study has documented an important association between inflammation and cognitive deficits (23). Clear data such as these are complicated by other findings; higher levels of C-reactive protein (CRP) are associated with the post-acute sequelae syndrome of COVID-19 (PASC), but not with cognition itself (24).

The COVID-19 pandemic has brought about a great deal of new research, but additional larger, well-designed studies are still needed (25) (26). This is particularly the case for COVID-19 studies addressing cognitive outcomes. Though informative, existing studies have had to rely on small sample sizes (27), a restricted set of predictor variables (28), a low number of analyzed cytokines (29), or a lack of objective psychiatric tools administered by trained professionals (e.g., neuropsychological battery, psychiatric interview) (30, 31). Thus, the aim of the present study was to analyze the potential impact of sociodemographic, clinical and biological factors on the long-term cognitive outcome of patients who survived moderate and severe forms of COVID-19. To this end, we utilized a large, hospital-based dataset to identify a cohort in the acute phase of the disease, and then completed a longitudinal follow-up of this cohort using a comprehensive set of clinical, neuropsychological and laboratory tools.

## Methods

### Study design and setting

This is a single center, cohort study conducted at *Hospital das Clínicas da Faculdade de Medicina da Universidade de São Paulo* (HCFMUSP), a university-based, tertiary medical facility that provided care for moderate and severe cases of the COVID-19 during the acute phase of the first wave of the pandemic, i.e., prior to the onset of vaccination protocols. The ‘HCFMUSP post-COVID-19 cohort’ was constituted to facilitate multidisciplinary studies addressing long-term medical, functional and neuropsychiatric outcomes among adults and elders who survived moderate or severe forms of COVID-19. Previously we reported a preliminary assessment of psychiatric and cognitive outcomes in an interim sample of 425 patients (i.e., half the size of the present test group) indicating high rates of mood and cognitive symptoms 6-11 months following infection (9). Details about the methodological protocol can also be found elsewhere (32).

This research protocol has been approved by the Ethics Committee at HCFMUSP (CAPPesq-HC), and registered at the Brazilian Registry of Clinical Trials (ReBEC) under the registration number 4.270.242 (RBR-8z7v5wc) and will be reported according to The Strengthening the Reporting of Observational Studies in Epidemiology (STROBE) Statement (33).

### Participants

All patients that were hospitalized at HCFMUSP for at least 24 hours due to moderate or severe forms of COVID-19 between March and August 2020 (n=3,753) were regarded as eligible for this ‘post-COVID-19 cohort’. From hospital registries, we ascertained all patients aged 18 years or older who were discharged from the hospital in this time period, excluding the deceased (n=1,052). Diagnostic confirmation was based on clinical presentation combined with either: a Polymerase Chain Reaction (PCR) tests to detect viral RNA or an enzyme-linked immunosorbent assays to detect the presence of anti-SARS-CoV-2 serum antibodies (in subjects for whom a RT-PCR test collected up to the 10th day of symptom onset was not available). These patients were contacted by telephone and enrolled in this follow-up study. In total, 1,957 patients were eligible for assessment (**Supplementary Figure 1**). Of these, some declined to participate (n=172); could not be contacted by telephone (n-=512); or did not show on their day of evaluation (n=62). An additional 12 potential participants were excluded due to a co-morbid dementia diagnosis; 26 were excluded for being evaluated in tele-appointments (i.e., lack of complete cognitive assessment) and 204 were excluded for having missing cognitive data. The missing cognitive data was due to a second COVID-19 wave that occurred during the protocol; meaning professionals were not readily available for research purposes. A further 157 individuals died and 102 were excluded for other reasons (e.g., missing data, lack of psychiatric protocol), leaving a total of 710 participants in the final sample.

### Assessment protocol

All participants signed consent forms and were assessed 6-11 months after hospital discharge (days; mean = 223; median = 202; SD 55.1) through structured interviews and assessment protocols administered by to an interdisciplinary medical team (from October/2020 to January/2021). Evaluation of mental state and global cognitive function was done in face-to-face interviews by a dedicated team of psychiatrists, psychologists, neuropsychologists, and undergraduate medical students. A set of data relative to the acute stage of the disease was retrieved from hospital charts and databases, providing baseline information on duration of hospital stay; requirement/duration of ICU care; requirement of orotracheal intubation, mechanical ventilation, or dialysis; and any available information about previous diagnoses, comorbidities, relevant clinical symptoms, and laboratory exams (after 72 hours of hospitalization). Severity of acute phase of COVID-19 was determined using the World Health Organization (WHO) criteria (34) ranging from 1 (less severe) to 4 (most severe). There was no systematic capture of neuropsychiatric and/or cognitive symptoms at baseline, except for recorded information about incident delirium, seizures, previous psychiatric disease (diagnosed by a specialist), or any signs suggestive of encephalopathy or cerebrovascular events during the acute phase of the disease. The complete description of the assessment protocol can be seen at **Supplementary Table 2**, but a brief description is provided below:

a) General Evaluation: Educational background (“no study ever” to post-graduation), Ethnicity, Socioeconomic Status (from the Brazilian Economic Classification Criterion – ABEP) ranging from A (best ranked) to E (worst ranked), Patient’s mental health, and occurrence of psychosocial/stressful events related to the COVID-19 pandemic (i.e., death of close family members; financial problems; and other relevant life-events or stressors).
b) Psychiatric Interview: Clinical Interview Schedule - Revised (CIS-R), Structured Clinical Interview for DSM-5 Disorders, Research Version (SCID-5-RV) for psychotic symptoms, Hospital Anxiety and Depression Scale (HAD), Ask Suicide-Screening Questions (ASQ), Post-Traumatic Stress Disorder Checklist (PCL-C), and Alcohol Use Disorder Identification Test (AUDIT).
c) Cognitive Assessment: Memory Complaint Scale (MCS) for both the patient and the closest informant. Here, we added a question to rank how the patients perceive themselves cognitively after COVID-19 (Similar or Better; Slightly Worse; or Much Worse). Temporal and Spatial Orientation (as obtained from the Mini-Mental State Examination, MMSE), Trail Making Test (TMT) – A, Verbal Fluency Test (VFT), Digit-Symbol Substitution Test (DDST), and the Consortium to Establish a Registry for Alzheimer’s Disease neuropsychological battery (CERAD), including Boston Naming Test, Word List Learning, Word List Recognition, Word List Recall, Constructional Praxis and Delayed Constructional Praxis.
d) Clinical Evaluation: An internal physician evaluated global health status (visual analogue scale), Clinical Frailty Scale (CFS) both pre- and post-COVID-19, International Physical Activity Questionnaire (IPAQ) – Short Version, presence of comorbidity (to calculate Charlson Score – (35)), Functional Assessment of Chronic Illness Therapy (FACIT) Scale to measure chronic fatigue, Smell and Taste function from visual analogue scale (0 to 100), body mass-index (BMI), pulse oximetry (to assess blood oxygen), and spirometry (for calculating forced vital capacity, FVC).
e) Cytokines: Plasma samples collected during follow-up of 389 out of the 710 participants were centrifugated and used for the analysis of 28 cytokines and chemokines. We employed the Human Cytokine/Chemokine Magnetic Bead Panel (Merck-Millipore, Cat. HCYTMAG-60K-PX30), according to the manufacturer’s instructions. 25 ul of serum were used. Analytes were detected on the Magpix^®^ instrument (Luminex Corp., Austin, TX 78727, USA). The calibration of the equipment was performed before use and followed all the manufacturer’s recommendations. The concentrations of cytokines and chemokines in each sample was calculated using a calibration curve obtained for each individual experiment with the diluent of each sample as vehicle, when necessary. When cytokines were not detected, we calculated the average of the detection limits for that factor and divided the value by the square root of two. The resulting value was assigned to that cytokine. To minimize inter-batch variation effects, cytokines were transformed using a R code (ComBat: Adjust for batch effects using an empirical Bayes framework in sva: Surrogate Variable Analysis. https://rdrr.io/bioc/sva/man/ComBat.html. Accessed 25 August 2022.).

### Statistical analysis

To facilitate data analysis, we transformed the objective cognitive assessments into Latent Cognitive Dimensions (LCD). First, we transformed the output of cognitive assessment tests into z-scores, as per cognitive domains. Second, we performed a Confirmatory Factor Analysis (CFA) to create LCD (see **Supplementary Figure 1**). We used CFA rather than Exploratory Factor Analysis (EFA) because previous literature supports the use of the cognitive dimensions measured here (36). Third, according to each factor loading we calculated an score for each of the following dimensions based on the indicated tests: a) Orientation (Spatial and Temporal Orientation from MMSE); b) Attention (TMT Time; TMT Errors; and DSST correct answers); c) Language (Boston Naming Test and Verbal Fluency – number of words); d) Episodic Memory (Word List Learning; Word List Recognition; and Word List Recall); e) Visuospatial Ability (Constructional Praxis and Delayed Constructional Praxis); and f) Global Cognition (composite score of all sub-dimensions).

For descriptive statistics, we calculated percentages, mean, median, standard deviation, and the upper and lower limits of the 95% confidence interval. We first conducted bivariate analysis between two groups (cytokines x non-cytokines) in order access potential selection bias. Then we performed a bivariate analysis (Pearson’s product-moment correlation, Kendall’s rank correlation tau, Student’s t test, One-way ANOVA, or Pearson’s Chi-squared test) between each sub-dimension and potential predictors, and those that reached p<.10 were selected for next steps. Linear regression with each cognitive dimension was performed using only independent variables from baseline. Any variable assessed in the follow-up was analysed using two different models; EFA (using Varimax Rotation) with continuous variables and cytokines (log-transformed) that reached significance with global cognitive dimension, to understand each PASC cluster; and Least Absolute Shrinkage and Selection Operator (LASSO) regression model with all cytokines, inflammatory markers, and follow-up potential associated and confounder variables. LASSO is reputed to be a very sensitive machine learning method for increasing the quality of prediction by “shrinking” regression coefficients (37), particularly when there are multiple independent variables potentially associated with distinct outcomes. Therefore, it is suitable for exploratory studies, due to its greater prediction accuracy as compared to other regression models (38). Each LASSO was repeated at least ten times in order to reduce any instability and possible effect of confounding factors.

## Results

Out of the 710 subjects that comprised our sample, 48.3% were female, with a mean age of 55 years (SD: 14.1). Regarding ethnicity, 65% identified as white, 8% as Black, 23.3% as Brown, and 0.4% as Yellow (further unknown). The WHO severity scores were normally distributed (Shapiro-Wilk p<.001), with a mean of 2.65 (SD: 1.12). The Charlson scores were also normally distributed (Shapiro-Wilk p<.001), with a mean of 3.10 (SD: 1.85). The mean duration of hospitalization was 17.6 days (SD 19.4). More than half of the patients (54.3%) required Intensive Care Unit (ICU) care (mean duration of ICU stay: 14.2 days, SD 13.8); 37.2% required orotracheal intubation (mean 10.8 days, SD: 8.77), and 12.5% required haemodialysis (mean 13.3 days, SD 11.2). Only 3.7% of participants reported a previous history of psychiatric disorders (i.e., any diagnosis prior to COVID-19 onset). That said, upon follow-up reassessment, we found a high prevalence of mood- and anxiety disorders (as indicated by the CIS-R schedule), including Generalized Anxiety Disorder (GAD, 14.6%), Depression (7.4%), and Common Mental Disorder (CMD, 30.2%). Concerning the self-assessment of cognitive performance (as indicated by participants or their caregivers), 49.3% endorsed being unaffected by COVID-19, 36.1% reported a slightly poorer overall performance in memory, and 14.6% reported being severely impacted, compared to their pre-COVID-19 status.

Persistent post-COVID-19 general health symptoms were often reported by participants, complying with the definition of PASC. Twenty percent of the patients reported having 1 or 2 persistent symptoms, whereas multiple symptoms were reported by the majority of the sample, i.e., 26% had 3-5 symptoms, and 45% of participants had more than 5 symptoms. Only 9% of the study group reported having no post-COVID-19 symptoms. Tiredness was the most frequent complaint (51%), followed by dizziness (36%), body aches (33%), dyspnoea (30%), severe muscular/joint pains (27%), nocturia (24%), chest pain (20%), cough (19%), oedema (17%), taste loss (16%), nasal obstruction (16%), skin problems (15%), smell loss (14%), tinnitus (14%), hearing loss (14%), abdominal pain (14%), appetite loss (13%), diarrhoea (6%), and nausea/vomiting (3%).

The following drugs were used for the pharmacological treatment of COVID-19 in the acute phase of the disease: vasopressors (5.5%), antiaggregant (19.3%), corticosteroids (60.6%), antiviral (33.9%), immunosuppressors (4.4%), antibiotics (92.6%), antifungals (6.5%), antiparasitic (7.1%), non-steroidal anti-inflammatories (23.2%), angiotensin-converting enzyme inhibitors (19.8%), angiotensin-II receptor antagonists (22.2%).

Bivariate analysis comparing two groups (cytokines x non-cytokines) found non-significant (p>.05) differences between groups regarding age, length of hospitalization, any cognitive dimension (general cognition, attention, orientation, episodic memory, language, visuospatial ability), comorbidity (Charlson severity), education level, socioeconomic status (ABEP), or previous psychiatric disease. A significant difference (p<.05) was found regarding sex (cytokines: male 46.9%, female 53.1%; non-cytokines: male 56.5%, female 43.5%) and WHO severity (cytokines: class 1, 9.3%, class 2, 38.4%, class 3, 3.6%, class 4, 48.7%; non-cytokines: class 1, 14.6%, class 2, 45%, class 3, 5%, class 4, 35.4%). CFA presented good fit (Standardized Root Mean Square Residual - SRMR = 0.04) reaching all five sub-dimensions. Bivariate analysis between cognitive dimensions and independent variables can be seen in **Supplementary Tables 3**, **4**. To test if our assumption was correct (i.e., individuals who claimed to be worse after COVID-19 were in fact cognitively worse), we performed two tests. First, patient and informant reports concerning patient cognition after COVID-19 were significantly associated (p<.001). Second, patients’ perceptions about changes in their cognitive state after COVID-19 were associated with their actual performance in objective tests addressing episodic memory (p=.002), orientation (p=.022), and global cognition (p=.026); but not for attention (p=.284), language (p=.184) and visuospatial ability (p=.539).

**Tables 1**–**5** presents linear regression models using baseline variables as predictors and each cognitive subdimension as outcomes. **Supplementary Table 5** presents a similar approach using global cognitive dimension as an outcome. We found statistically significant relationships between cognitive outcomes (orientation and attention, in particular) and socio-demographic variables (i.e., sex and education level).

**Table 1:** Linear Regression between baseline variables and Orientation.

	Coefficient	SE	Lower CI	Upper CI	p-value
(Intercept)	-0.374	0.362	-1.085	0.336	0.301
Age	-0.004	0.003	-0.011	0.002	0.213
Sex [Male]	0.136	0.067	0.005	0.268	0.042
Ethnicity [Yellow]	0.570	0.496	-0.403	1.544	0.250
Ethnicity [Black]	0.097	0.121	-0.142	0.335	0.427
Ethnicity [Brown]	-0.079	0.079	-0.234	0.076	0.318
ABEP [B1]	0.115	0.240	-0.356	0.586	0.631
ABEP [B2]	0.214	0.223	-0.225	0.653	0.339
ABEP [C1]	0.186	0.223	-0.252	0.623	0.405
ABEP [C2]	0.052	0.227	-0.393	0.498	0.818
ABEP [D-E]	0.037	0.249	-0.451	0.525	0.882
Education [Uncompleted Elementary/Middle School]	0.426	0.180	0.073	0.779	0.018
Education [Completed Elementary/Middle School]	0.818	0.201	0.422	1.214	< 0.001
Education [Uncompleted High School]	0.525	0.212	0.108	0.942	0.014
Education [Completed High School]	0.801	0.194	0.421	1.182	< 0.001
Education [Uncompleted Undergraduation]	0.826	0.234	0.367	1.285	< 0.001
Education [Completed Undergraduation]	0.866	0.224	0.425	1.306	< 0.001
Education [Post-graduation]	0.927	0.264	0.408	1.445	< 0.001
Charlson Severity Score	-0.006	0.024	-0.053	0.040	0.789
Previous psychiatric disease [Yes]	-0.373	0.166	-0.700	-0.046	0.026
Severity WHO [2]	0.110	0.101	-0.088	0.307	0.277
Severity WHO [3]	0.176	0.174	-0.167	0.518	0.315
Severity WHO [4]	0.098	0.112	-0.123	0.319	0.384
Basal C-protein	0.000	0.000	-0.001	0.001	0.560
Basal D-dimer	0.000	0.000	0.000	0.000	0.577
Pre-COVID-19 frailty	-0.100	0.032	-0.162	-0.039	0.002
IPAQ [Irregularly Active]	0.112	0.086	-0.057	0.282	0.194
IPAQ [Active]	0.105	0.079	-0.050	0.260	0.182
IPAQ [Very Active]	0.216	0.176	-0.129	0.560	0.220

**Table 2:** Linear Regression between baseline variables and Attention.

	Coefficient	SE	Lower CI	Upper CI	p-value
(Intercept)	0.447	0.447	-0.432	1.325	0.318
Age	-0.023	0.004	-0.031	-0.015	< 0.001
Sex [Male]	0.187	0.083	0.025	0.350	0.024
Ethnicity [Yellow]	0.075	0.601	-1.105	1.256	0.900
Ethnicity [Black]	0.029	0.150	-0.265	0.324	0.845
Ethnicity [Brown]	-0.134	0.101	-0.332	0.064	0.184
ABEP [B1]	-0.077	0.291	-0.648	0.495	0.793
ABEP [B2]	0.056	0.272	-0.478	0.589	0.837
ABEP [C1]	-0.029	0.270	-0.559	0.500	0.913
ABEP [C2]	-0.223	0.276	-0.765	0.318	0.418
ABEP [D-E]	-0.305	0.302	-0.899	0.288	0.312
Education [Uncompleted Elementary/Middle School]	0.519	0.228	0.070	0.968	0.023
Education [Completed Elementary/Middle School]	1.093	0.253	0.595	1.592	< 0.001
Education [Uncompleted High School]	1.298	0.267	0.772	1.823	< 0.001
Education [Completed High School]	1.676	0.243	1.198	2.154	< 0.001
Education [Uncompleted Undergraduation]	2.228	0.293	1.653	2.803	< 0.001
Education [Completed Undergraduation]	2.138	0.280	1.589	2.688	< 0.001
Education [Post-graduation]	2.413	0.325	1.774	3.052	< 0.001
Charlson Severity Score	-0.089	0.029	-0.146	-0.032	0.002
Previous psychiatric disease [Yes]	-0.239	0.192	-0.616	0.138	0.213
Severity WHO [2]	0.085	0.123	-0.156	0.327	0.488
Severity WHO [3]	-0.094	0.218	-0.522	0.334	0.667
Severity WHO [4]	0.022	0.138	-0.248	0.293	0.872
Basal C-protein	0.000	0.000	-0.001	0.001	0.977
Basal D-dimer	0.000	0.000	0.000	0.000	0.916
Pre-COVID-19 frailty	-0.075	0.040	-0.153	0.003	0.060
IPAQ [Irregularly Active]	0.099	0.106	-0.111	0.308	0.355
IPAQ [Active]	0.110	0.097	-0.080	0.300	0.255
IPAQ [Very Active]	0.098	0.212	-0.319	0.515	0.645

**Table 3:** Linear Regression between baseline variables and Language.

	Coefficient	SE	Lower CI	Upper CI	p-value
(Intercept)	-0.068	0.363	-0.781	0.645	0.851
Age	-0.013	0.003	-0.020	-0.006	< 0.001
Sex [Male]	0.223	0.068	0.089	0.356	0.001
Ethnicity [Yellow]	-0.020	0.504	-1.009	0.969	0.968
Ethnicity [Black]	0.062	0.122	-0.178	0.302	0.614
Ethnicity [Brown]	-0.201	0.080	-0.358	-0.044	0.012
ABEP [B1]	-0.271	0.244	-0.750	0.207	0.266
ABEP [B2]	-0.081	0.229	-0.531	0.369	0.724
ABEP [C1]	-0.286	0.226	-0.731	0.158	0.207
ABEP [C2]	-0.154	0.232	-0.609	0.301	0.506
ABEP [D-E]	-0.046	0.251	-0.539	0.447	0.855
Education [Uncompleted Elementary/Middle School]	0.607	0.176	0.262	0.952	< 0.001
Education [Completed Elementary/Middle School]	1.124	0.196	0.739	1.508	< 0.001
Education [Uncompleted High School]	1.233	0.210	0.821	1.646	< 0.001
Education [Completed High School]	1.319	0.190	0.946	1.691	< 0.001
Education [Uncompleted Undergraduation]	1.678	0.231	1.224	2.131	< 0.001
Education [Completed Undergraduation]	1.779	0.221	1.344	2.213	< 0.001
Education [Post-graduation]	1.894	0.260	1.382	2.405	< 0.001
Charlson Severity Score	-0.065	0.024	-0.112	-0.018	0.007
Previous psychiatric disease [Yes]	-0.283	0.180	-0.640	0.073	0.118
Severity WHO [2]	0.136	0.102	-0.063	0.336	0.181
Severity WHO [3]	-0.107	0.181	-0.462	0.249	0.555
Severity WHO [4]	0.139	0.113	-0.084	0.361	0.221
Basal C-protein	0.000	0.000	-0.001	0.001	0.515
Basal D-dimer	0.000	0.000	0.000	0.000	0.854
Pre-COVID-19 frailty	-0.046	0.032	-0.110	0.017	0.154
IPAQ [Irregularly Active]	0.193	0.088	0.021	0.365	0.028
IPAQ [Active]	0.123	0.080	-0.033	0.280	0.122
IPAQ [Very Active]	0.068	0.176	-0.277	0.413	0.698

**Table 4:** Linear Regression between baseline variables and Episodic Memory.

	Coefficient	SE	Lower CI	Upper CI	p-value
(Intercept)	2.051	0.686	0.704	3.399	0.003
Age	-0.038	0.006	-0.051	-0.025	< 0.001
Sex [Male]	0.078	0.129	-0.175	0.332	0.544
Ethnicity [Yellow]	0.030	0.948	-1.831	1.892	0.975
Ethnicity [Black]	-0.161	0.231	-0.614	0.293	0.487
Ethnicity [Brown]	-0.163	0.152	-0.462	0.136	0.285
ABEP [B1]	-0.939	0.457	-1.837	-0.041	0.040
ABEP [B2]	-0.730	0.425	-1.566	0.105	0.087
ABEP [C1]	-0.729	0.422	-1.558	0.099	0.084
ABEP [C2]	-0.957	0.431	-1.803	-0.111	0.027
ABEP [D-E]	-1.137	0.470	-2.059	-0.214	0.016
Education [Uncompleted Elementary/Middle School]	0.412	0.331	-0.238	1.063	0.214
Education [Completed Elementary/Middle School]	1.011	0.368	0.288	1.733	0.006
Education [Uncompleted High School]	1.279	0.396	0.502	2.056	0.001
Education [Completed High School]	1.323	0.359	0.617	2.029	< 0.001
Education [Uncompleted Undergraduation]	1.226	0.435	0.372	2.079	0.005
Education [Completed Undergraduation]	1.410	0.420	0.584	2.235	< 0.001
Education [Post-graduation]	2.199	0.491	1.234	3.163	< 0.001
Charlson Severity Score	-0.128	0.045	-0.216	-0.039	0.005
Previous psychiatric disease [Yes]	-0.457	0.363	-1.180	0.267	0.212
Severity WHO [2]	0.388	0.193	0.010	0.766	0.044
Severity WHO [3]	0.394	0.337	-0.268	1.056	0.243
Severity WHO [4]	0.141	0.216	-0.282	0.565	0.512
Basal C-protein	0.000	0.001	-0.001	0.002	0.618
Basal D-dimer	0.000	0.000	0.000	0.000	0.352
Pre-COVID-19 frailty	-0.034	0.061	-0.155	0.086	0.574
IPAQ [Irregularly Active]	0.288	0.166	-0.038	0.614	0.084
IPAQ [Active]	0.174	0.150	-0.121	0.470	0.246
IPAQ [Very Active]	0.457	0.332	-0.194	1.108	0.169

**Table 5:** Linear Regression between baseline variables and Visuospatial Ability.

	Coefficient	SE	Lower CI	Upper CI	p-value
(Intercept)	-0.491	0.505	-1.482	0.500	0.331
Age	-0.020	0.005	-0.029	-0.011	< 0.001
Sex [Male]	0.127	0.094	-0.058	0.312	0.179
Ethnicity [Yellow]	1.530	0.714	0.128	2.932	0.032
Ethnicity [Black]	0.129	0.169	-0.202	0.460	0.445
Ethnicity [Brown]	-0.141	0.111	-0.359	0.076	0.203
ABEP [B1]	0.357	0.336	-0.302	1.016	0.288
ABEP [B2]	0.237	0.314	-0.379	0.854	0.450
ABEP [C1]	0.083	0.312	-0.529	0.695	0.789
ABEP [C2]	0.013	0.318	-0.611	0.638	0.966
ABEP [D-E]	0.138	0.348	-0.546	0.821	0.693
Education [Uncompleted Elementary/Middle School]	1.131	0.245	0.650	1.613	< 0.001
Education [Completed Elementary/Middle School]	1.653	0.273	1.117	2.188	< 0.001
Education [Uncompleted High School]	1.661	0.293	1.086	2.236	< 0.001
Education [Completed High School]	2.020	0.266	1.499	2.542	< 0.001
Education [Uncompleted Undergraduation]	2.381	0.321	1.750	3.012	< 0.001
Education [Completed Undergraduation]	2.494	0.310	1.886	3.103	< 0.001
Education [Post-graduation]	2.751	0.362	2.040	3.462	< 0.001
Charlson Severity Score	-0.011	0.033	-0.077	0.054	0.738
Previous psychiatric disease [Yes]	-0.223	0.234	-0.686	0.240	0.343
Severity WHO [2]	0.035	0.141	-0.241	0.311	0.801
Severity WHO [3]	-0.236	0.246	-0.720	0.248	0.339
Severity WHO [4]	0.072	0.157	-0.235	0.380	0.645
Basal C-protein	0.000	0.001	-0.001	0.001	0.430
Basal D-dimer	0.000	0.000	0.000	0.000	0.531
Pre-COVID-19 frailty	-0.114	0.046	-0.203	-0.025	0.013
IPAQ [Irregularly Active]	0.148	0.122	-0.091	0.387	0.226
IPAQ [Active]	0.005	0.110	-0.211	0.222	0.961
IPAQ [Very Active]	0.170	0.244	-0.310	0.649	0.487

All factors were significantly associated with higher educational profile as a protective factor, whereas male sex, in turn, was a protective factor for all but visuospatial ability and episodic memory. Older age was significantly associated as a risk factor for all variable except orientation. Comorbidity (Charlson severity score) was associated with all but orientation and visuospatial ability as risk factors. Several other variables were associated with specific independent variables. First, *orientation* was significantly associated previous psychiatric disease and pre-COVID-19 frailty as risk factors. A*ttention* was not associated with any other variable. Brown ethnicity was associated with *Language*, as a risk factor, which in turn, was also associated with low physical activity as a protective factor. *Episodic memory* was associated with COVID-19 severity and low socioeconomic status as a risk factors. *Visuospatial ability*, in turn, was associated with pre-COVID-19 frailty as risk factor; and finally, *Global cognition* was associated with Brown ethnicity and pre-COVID-19 frailty as risk factors and low physical exercise as a protective factor.

**Supplementary Table 6** presents a model to better understand PASC clusters in our sample. We found five different clusters with a total explained variance of 58.6% (i.e., only loadings > 0.4):

*factor 1*: IL4, IL5, IL1-RA, IL1-alpha, IL13, IL6, IL15, and TNF-beta
*factor 2:* VEGF, IL7, IFN-alpha2, G-CSF, IL1-RA, and IL15
*factor 3:* chronic fatigue, MCS patient, depression, PTSD, and anxiety
*factor 4:* orientation, attention, language, episodic memory, and visuospatial ability
*factor 5:* smell and taste.

Finally, LASSO regression with all follow-up variables, inflammatory markers and cytokines did not find any significant variables associated with any cognitive sub-dimension or the global cognitive dimension.

## Discussion

Here we present a diverse set of sociodemographic, clinical and biological variables associated with cognitive impairment in a cohort of survivors of moderate and severe forms of SARS-CoV-2 infection. First, we identified sociodemographic variables associated with poor cognitive performance; such as older age, female sex, ethnicity endorsed as Brown, and a lower educational profile. Second, clinical variables associated with poorer global cognition were: high comorbidity, low physical exercise, and a more severe frailty pre-COVID-19. Third, though we identified five clusters associated with post-COVID-cognitive disturbances, cognition appeared as a separate and individual factor. And fourth, a LASSO analysis did not identify any clinical or biological (inflammatory markers and cytokines during follow-up) associated with poorer cognitive performance for any cognitive dimension, or for global cognition in general.

There is no debate that post-COVID-19 patients can develop a persistent spectrum of cognitive disturbances (7), which some authors liken to an Alzheimer’s Disease (AD)-type cognitive impairment (39). In fact, recent studies have pointed out several neuropathological similarities of PASC Cognitive Syndrome with AD (26); including, numerous elevated AD marker genes (e.g., FERMT2, HLA-DRB1, GNA15, STAB1, ICA1L, COLGALT1, TNFAIP2, ITGAM, VASP, IDLIA, PVR, TECPR1) (39), several circulatory biomarkers (i.e. GFAP, NFL, P-tau 181, UCH, NSE, and S100B) (26), and the presence of Apolipoprotein E ϵ4 allele (APOE4) (40, 41), consistent with other reports (6). Curiously, in LASSO regression modelling, we did not find a link between inflammatory cytokines and cognitive disturbances, as has been previously reported (7, 14, 42). These data suggest that future research might focus on additional AD biomarkers, rather than solely on inflammatory cytokines.

Although LASSO regression modelling did not find significant relationships between cognitive impairments and cytokine levels, bivariate analysis between cognitive sub-dimensions (**Supplementary Table 3**) suggested several cytokines that may be involved in cognitive function and may warrant further investigation. In particular, IL-1RA, IL-7, and G-CSF were associated with attention, language, episodic memory, and cognition dimensions. These factors have previously been implicated in cognitive function. For example, in adults with multiple sclerosis, a higher serum concentration of the anti-inflammatory marker IL-1RA was associated with better social-cognitive functioning (43). A study of 42 adults with bipolar disorder found that IL-7 levels were significantly associated with measures of cognition, showing higher levels in the cognitively unimpaired group and a positive correlation with cognitive performance (44). Further, preclinical studies indicate that treatment with G-CSF, a growth factor involved in neuroprotection and plasticity, may contribute to improved cognitive function in a model of traumatic brain injury (45). However, the potential role of IL-1RA, IL-7, and G-CSF in long COVID outcomes is unknown. In a previous study, IL-1RA and IL-7 were reported to be higher in the plasma of patients who recovered from COVID-19 compared to healthy controls and patients with acute COVID-19, however, G-CSF did not differ between control patients and patients recovered from COVID-19 (46). IL-1RA is an anti-inflammatory cytokine due to its IL-1 antagonistic actions inhibiting IL-1α and IL-1β signaling. G-CSF has neuroprotective properties as it inhibits apoptosis and inflammation in the brain, and also stimulates neurogenesis (47).

We acknowledge that the lack of statistically significant associations between inflammatory markers and cognitive impairment was unexpected. This negative finding might be explained by several reasons: first, the effect of confounding variables, such as psychiatric, fatigue and pulmonary symptoms. This in line with a previous study conducted by our group, in which we found a significant association between “long-COVID” (defined as a latent dimension) and higher levels of C-reactive protein and d-dimer, but not with any specific psychiatric or cognitive symptom (24). This isan interesting finding giving that in Busatto et al., latent PASC is dominated by fatigue, insomnia, psychiatric and cognitive symptoms. We hypothesize that the interaction among all symptoms increase the strength of association, especially in a sample of severe individuals (post-hospitalized); and highlights that the lack of association does not exclude the potential role of inflammation impacting the cognition of these individuals. Second, the fact that cytokines were determined only in a subset (n=389) of the total follow-up sample (n=710). The inclusion of participants in this sub-sample was not random; rather, it prioritized the occurrence of general PASC symptoms (including, but not restricted to, cognitive symptoms), rendering the analysis prone to selection bias. Third, the LASSO regression per se, which may have supressed the significance of weaker associations between variables through the “shrinking” process. Finally, the fact that cytokines were not determined at baseline, precluding its comparison with follow-up values. This may be particularly relevant in the light of the frequent prescription of corticosteroids to post-COVID-19 patients, along with studies showing that this intervention may actually attenuate the so-called “cytokine storm” (48).

Moreover, we did not observe a significant association between observed clinical (i.e., pulmonary disorder, fatigue, smell and taste impairment, and COVID-19 severity) and psychiatric disorders (i.e. depression, GAD, PTSD, and CMD) and cognitive disturbances. The EFA data were consistent with this notion; cognitive impairment is a separate and specific cluster of the PASC syndrome. Instead, after controlling for multiple variables, we found that the presence of higher comorbidity and more severe frailty pre-COVID-19, as well as lower physical exercise in the weeks prior to the follow-up assessment, predicted poorer cognitive performance. The first two variables (comorbidity and frailty) have been previously discussed (7, 9), however, the latter variable (physical exercise) is a new finding that might be an important target for neuropsychological rehabilitation techniques in PASC patients. Such a strategy would be consistent with the protective effect of physical exercise observed in individuals with AD (49), particularly those who carry the Apolipoprotein E ϵ4 allele (50) that modulates AD biomarkers (51).

One of the most robust findings in the present study was the observed relationship between different sociodemographic phenotypes and cognitive decline in long-COVID-19. Older age, female sex, ethnicity endorsed as Brown, and a lower educational profile predicted lower cognitive performance, with educational profile having the greatest effect size. Several other studies have reported poorer cognitive performance in PASC individuals who are older and female (7). A lower educational profile comprises one of the main factors related to cognitive reserve and might be one of the main risk factors following acute stressors (52, 53) such as COVID-19 (54, 55). It is noteworthy that, in the present sample, the significant association between cognitive performance and lower educational profile was not accompanied by an association with socioeconomic status, given the fact that these two variables are often intertwined. We hypothesize that this lack of association in the present analysis may be due to a floor effect, given that 73.81% of our sample was raked as pertaining to lower socioeconomic classes (C-E).

It is important to point out to some limitations. First, we did not have a pre-COVID cognitive assessment. However, we did demonstrate that patients who claimed that their mental faculties were worse post-SARS CoV-2 infection, were in fact cognitively worse. Other qualifiers of the present study include: a) this is a single-centre study from a single country, which might limit its generalizability, b) our cohort is made up of relatively older individuals, which might increase the likelihood of a ceiling effect, reducing potentially significant associations, and c) cytokines were analysed 6-11 months after COVID-19 infection and not in acute phase, which could have influenced our results. However, our stated aim was to analyse long-term inflammatory markers, in order to fill this gap in literature.

In summary, here we highlight the importance of several sociodemographic characteristics that might protect against cognitive impairment following SARS-CoV-2 infection. Our data do not find a prominent role for clinical status (both during acute and long-stage of COVID-19) or inflammatory background (also during acute and long-stage of COVID-19) to explain cognitive deficits following infection. These findings will require further validation by other centres. These results also point to possible interventions for cognitive impairment following COVID-19 (e.g., exercise) that future studies might address.

## Data availability statement

The original contributions presented in the study are included in the article/**Supplementary Material**. Further inquiries can be directed to the corresponding author.

## Ethics statement

The studies involving human participants were reviewed and approved by Ethics Committee of Hospital das Clínicas da Universidade de São Paulo. The patients/participants provided their written informed consent to participate in this study.

## Author contributions

The authors confirm contribution to the paper as follows: study conception and design: RD, CR, AS, LT, PP, EC-N, JK, GC, MS, BG, SM, HS, RN, EM, GB, OF. Data collection: RD, CR, AS. Analysis and interpretation of results: RD, AS, JL, LT, PP, EC-N, JK, GC, MS, BG, SM, HS, RN, EM, GB, OF. Draft manuscript preparation: RD, CR, AS, JL, LT, PP, EC-N, JK, GC, MS, BG, SM, HS, RN, EM, GB, OF. All authors reviewed the results and approved the final version of the manuscript.

## HCFMUSP COVID-19 Study Group

Edivaldo M. Utiyama, Aluisio C. Segurado, Beatriz Perondi, Anna Miethke-Morais, Amanda C. Montal, Leila Harima, Solange R. G. Fusco, Marjorie F. Silva, Marcelo C. Rocha, Izabel Marcilio, Izabel Cristina Rios, Fabiane Yumi Ogihara Kawano, Maria Amélia de Jesus, Ésper G. Kallas, Carolina Carmo, Clarice Tanaka, Julio F. M. Marchini, Carlos R. Carvalho, Juliana C. Ferreira, Anna Sara Levin, Maura Salaroli Oliveira, Thaís Guimarães, Carolina dos Santos Lázari, Alberto José da Silva Duarte, Ester Sabino, Marcello M. C. Magri, Tarcisio E. P. Barros-Filho, Maria Cristina Peres Braido Francisco.
