# Supplementary material for: Cognitive impairment in long-COVID and its association with persistent dysregulation in inflammatory markers
Source: Front Immunol. 2023 May 23;14:1174020. doi: 10.3389/fimmu.2023.1174020 (PMC10242059; doi:10.3389/fimmu.2023.1174020)
Supplement: Supplementary file 1 [file DataSheet_1.docx]

**Supplementary Table 1.** Flow-chart of evaluated individuals in cognitive assessment of the cohort

**Supplementary Table 2.** Instruments Used in the Follow-up Evaluation

| **Scale** | **Characteristic** | **References** |
| --- | --- | --- |
| **A. Diagnostic Interview** |  |  |
| Clinical Interview Schedule - Revised (CIS-R) | It is a structured psychiatric interview developed by Lewis et al. (1992) and culturally adapted to the Brazilian population by Nunes et al. (2011). It consists of 14 sections addressing: somatic symptoms; fatigue; sleep problems; irritability; physical health worries; depression; depressive ideas; worry; anxiety; phobias; panic; compulsive behaviors; obsessive thoughts; forgetfulness/concentration problems, yielding 5 psychiatric categories based on ICD-10: generalized anxiety disorder, depressive episode, all phobias (agoraphobia, social phobia, and simple phobia), obsessive-compulsive disorder, and panic disorder. The diagnosis of a common mental disorder can be established if the participant scores ≥12 in the sum of all 14-dimensional symptoms. It presents good psychometric characteristics even when compared with more robust instruments such as the Structured Clinical Interview for DSM-5 Disorders (SCID-5). | Lewis G, Pelosi AJ, Araya R, Dunn G. Measuring psychiatric disorder in the community: a standardized assessment for use by lay interviewers. Psychological medicine 1992; 22(2): 465-86. Nunes MA, Alves MGdM, Chor D, Schmidt MI, Duncan BB. Adaptação transcultural do CIS-R (Clinical Interview Schedule - Revised Version) para o português no estudo longitudinal de saúde do adulto (ELSA). 2012 2012; 31(4). Jordanova V, Wickramesinghe C, Gerada C, Prince M. Validation of two survey diagnostic interviews among primary care attendees: a comparison of CIS-R and CIDI with SCAN ICD-10 diagnostic categories. Psychological medicine 2004; 34(6): 1013-24. Pez O, Gilbert F, Bitfoi A, et al. Validity across translations of short survey psychiatric diagnostic instruments: CIDI-SF and CIS-R versus SCID-I/NP in four European countries. Soc Psychiatry Psychiatr Epidemiol 2010; 45(12): 1149-59. |
| Structured Clinical Interview for DSM-5 Disorders, Research Version (SCID-5-RV) | The assessment of psychotic symptoms was done with the aid of an excerpt of the SCID-5-RV schedule, i.e., Module B, Psychotic and Associated Symptoms (items B2 to B19). The SCID-5-RV is a semi-structured psychiatric interview that follows the diagnostic criteria established by the American Psychiatric Association (APA)’s Diagnostic and Statistical Manual for Mental Disorders, 5th Edition (DSM-5). Given that the target population comprised subjects with no (a priori) previous history of psychotic disorders, and to render the interpretation of responses easier for the examiners, this assessment was limited to nineteen objective questions yielding yes/no answers (i.e., symptom present or absent). Thirteen of those questions address different types of delusions, while the remaining six assess auditory, visual, tact, taste, olfactory and somatic hallucinations. | First M, Williams J, Karg R, Spitzer R. Structured Clinical Interview for DSM-5 Disorders, Clinician Version (SCID-5-CV). . Arlington, VA: American Psychiatric Association; 2016. |
| **B. Self-Report Measures** |  |  |
| Hospital Anxiety and Depression Scale (HAD) | It is a self-assessment scale developed by Zigmond and Snaith (1983) and validated to Brazilian clinical population by Botega et al. (1995). The HAD is a widely used reliable instrument to determine the levels of anxiety, depression and emotional disorders in hospitalized and post-hospitalized patients, due to its focus on psychological rather than somatic symptoms of depression. The scale is composed of 14 questions scored from 0-3, subdivided in two domains (anxiety and depression) of seven questions each. Total score ranges from 0 to 21, higher scores indicating more severe symptoms. We used cut off of ≥8 for both subscales, which supposedly yields 82% sensitivity for the identification major depressive disorder (MDD) and 78% for generalized anxiety disorder (GAD), along with 74% specificity for MDD and GAD. | Zigmond AS, Snaith RP. The hospital anxiety and depression scale. Acta Psychiatr Scand 1983; 67(6): 361-70. Botega NJ, Bio MR, Zomignani MA, Garcia Jr C, Pereira WAB. Transtornos do humor em enfermaria de clínica médica e validação de escala de medida (HAD) de ansiedade e depressão. Revista de Saúde Pública 1995; 29: 359-63. Brennan C, Worrall-Davies A, McMillan D, Gilbody S, House A. The Hospital Anxiety and Depression Scale: A diagnostic meta-analysis of case-finding ability. Journal of Psychosomatic Research 2010; 69(4): 371-8. |
| Ask Suicide-Screening Questions (ASQ) | The ASQ is a four-item self-report questionnaire to screen for suicide risk. It evaluates the occurrence of suicidal ideation in the previous four weeks, in addition to any previous attempts. The questions address the ‘wish to die’, the feeling of ‘leaving one’s family better off if dead’, the presence of suicidal thoughts, and any previous suicide attempts. The first three answers range from never to daily (0-3), implying the risk of suicide. The scale further estimates the number of suicide attempts, if any, in the previous year. Studies in pediatric emergency settings indicated good psychometric properties, with 96.9% sensitivity, 87.6% specificity, and negative predictive value (NPV) of 99.7%.39 The ASQ has also been validated for use among adults, representing a good tool for the screening of suicidal risk behavior with specificity and NPV rates of 89% and 100% respectively. For this study, we used a score (sum of questions 1, 2, 3, 4 and 6) in order to produce a continuous variable. | Horowitz LM, Bridge JA, Teach SJ, et al. Ask Suicide-Screening Questions (ASQ): a brief instrument for the pediatric emergency department. Arch Pediatr Adolesc Med 2012; 166(12): 1170-6. Horowitz LM, Snyder DJ, Boudreaux ED, et al. Validation of the Ask Suicide-Screening Questions for Adult Medical Inpatients: A Brief Tool for All Ages. Psychosomatics 2020; 61(6): 713-22. |
| Post-Traumatic Stress Disorder Checklist (PCL-C) | It is an instrument developed for the assessment of PTSD, based on DSM-III-R diagnostic criteria that has a validated Brazilian Portuguese version. The scale takes into account the severity of symptoms reported by the subject in the previous month, utilizing a grading scale ranges from ‘nothing’ to ‘extremely’ (1-5). For PTSD diagnosis, the patient needs to have at least moderate symptoms (score ≥3) in one or more criteria listed in cluster B, three in cluster C, and two in cluster D. Raters were instructed to score only if suspected PTSD symptoms occurred after COVID-19 onset. | Weathers F, Litz B, Herman D, Huska JA, Keane T. PTSD Checklist: Reliability, validity, and diagnostic utility. Proceedings of the 9th Annual Meeting of the International Society for Traumatic Stress Studies (ISTSS) 1993. Berger W, Mendlowicz MV, Souza WF, Figueira I. Equivalência semântica da versão em português da Post-Traumatic Stress Disorder Checklist - Civilian Version (PCL-C) para rastreamento do transtorno de estresse pós-traumático. Revista de Psiquiatria do Rio Grande do Sul 2004; 26: 167-75. |
| Alcohol Use Disorder Identification Test (AUDIT) | The AUDIT is a widely used instrument developed by the World Health Organization to estimate Alcohol Use Disorder (AUD). It is a comprehensive 10-item self-report screening tool, with total score ranging from 0 to 40, indicating ‘low risk’ (0-7), ‘increasing risk’ (8-15), ‘higher risk’ (16-19) and ‘possible dependence’ (20 or more). A Brazilian Portuguese version has been validated for use in urban populations, with good psychometric proprieties. | Reinert DF, Allen JP. The Alcohol Use Disorders Identification Test (AUDIT): A review of recent research. Alcoholism: Clinical and Experimental Research 2002; 26(2): 272-9. World Health O. AUDIT: the Alcohol Use Disorders Identification Test : guidelines for use in primary health care / Thomas F. Babor ... [‎‎et al.]‎‎. 2nd ed ed. Geneva: World Health Organization; 2001. Lima CT, Freire AC, Silva AP, Teixeira RM, Farrell M, Prince M. Concurrent and construct validity of the audit in an urban brazilian sample. Alcohol Alcohol 2005; 40(6): 584-9. |
| **C. Cognitive Assessment** |  |  |
| Memory Complaint Scale (MCS) | The MSC carries out a systematic search for memory complaints. It is composed of seven self-reported items with graded responses where higher scores indicate greater intensity (0, 1 and 2). The memory complaints are ranked as ‘absent’ (0-2), ‘mild’ (3-6), ‘moderate’ (7-10) and ‘severe’ (11-14). This instrument explores the frequency and the degree to which the memory complaints impact on daily activities; compares the current memory to that of a younger age, and to that of others within the same age range. The scale has two identical versions (A and B), the latter dedicated to capture the informant’s report (if available) about the subject’s memory complaints. Previous research suggested that subjective memory complaints may be a proxy of poor cognitive function in older adults. After completion of the MCS schedule, participants were additionally asked to rank their overall memory performance in the light of COVID-19. | Vale FAC, Balieiro-Jr AP, Silva-Filho JH. Memory complaint scale (MCS): Proposed tool for active systematic search. Dementia & Neuropsychologia 2012; 6: 212-8. Amariglio RE, Townsend MK, Grodstein F, Sperling RA, Rentz DM. Specific subjective memory complaints in older persons may indicate poor cognitive function. J Am Geriatr Soc 2011; 59(9): 1612-7. |
| *Digit Symbol Substitution Test (DDST)* | The DDST is a widely used test in neuropsychology. It consists in a series of numbers and symbols where participants are asked to fill out blank spaces in two minutes. In our test, we asked to fill out the respective number looking for each specific symbol. The score consists of summing the right answers (right numbers) in two minutes. | Jaeger J. Digit Symbol Substitution Test: The Case for Sensitivity Over Specificity in Neuropsychological Testing. Journal of clinical psychopharmacology. 2018;38(5):513-9. |
| Temporal and Spatial Orientation of Mini Mental State Examination (MMSE) | This study utilizes the temporal and spatial orientation section of the mini-mental state examination (MMSE) composed of 10 questions for which answers were classified as either correct or incorrect. It is asked that the patient specify the day of the week, the day of the month, which month, which year and at what time the interview is being conducted. The spatial orientation is determined by assessing if the patient is able to correctly name the following items: the specific location the interview is being conducted at; the building he or she is in; the neighborhood or any close by streets; the country and the state. The choice for this part of the instrument was meant to discriminate severe forms of dementia, which could impact our final outcome and interpretation. | Bernard BA, Goldman JG. MMSE - Mini-Mental State Examination. In: Kompoliti K, Metman LV, eds. Encyclopedia of Movement Disorders. Oxford: Academic Press; 2010: 187-9. |
| Consortium to Establish a Registry for Alzheimer's Disease neuropsychological battery (CERAD) | Developed by the Consortium to Establish a Registry for Alzheimer’s Disease and adapted to Brazilian population by Bertolucci et al., it consists in a large cognitive battery assessing different cognitive domains. We uded the following instruments from CERAD: Boston Naming Test, Word List Learning, Word List Recall, Constructional Praxis and Delayed Constructional Praxis. | Welsh K, Butters N, Hughes J, Mohs R, Heyman A. Detection of abnormal memory decline in mild cases of Alzheimer's disease using CERAD neuropsychological measures. Arch Neurol. 1991;48(3):278-81.  Bertolucci PHF, Okamoto IH, Brucki SMD, Siviero MO, Toniolo Neto J, Ramos LR. Applicability of the CERAD neuropsychological battery to Brazilian elderly. Arquivos de Neuro-Psiquiatria. 2001;59:532-6. |
| **D. Clinical Assessment** |  |  |
| Functional Assessment of Chronic Illness Therapy (FACIT) Fatigue Scale | FACIT is a self-report scale developed to measure chronic fatigue following clinical or psychiatric ilnessess. It is a 13-item scale, with a 4-point likert (ranging from 4 = not at all fatigued to 0 = very much fatigued). It demonstrated good psychometric proprieties and a translated version has been used in Brazilian samples. | Yellen SB, Cella DF, Webster K, Blendowski C, Kaplan E. Measuring fatigue and other anemia-related symptoms with the Functional Assessment of Cancer Therapy (FACT) measurement system. J Pain Symptom Manage. 1997;13(2):63-74.  Bianchi WA, Elias FR, Pinheiro Gda R, et al. Analysis of the association of fatigue with clinical and psychological variables in a series of 371 Brazilian patients with rheumatoid arthritis. Rev Bras Reumatol. 2014;54(3):200-207. |
| Clinical Frailty Scale (CFS) | The CFS was developed for use in the Canadian Study of Health and Aging (CSHA), and described by Rockwood et al. It is composed of a 7-item scale that clinicians have to ascertain for the vulnerability of each patient, where 1 represents very fit (robust, active, energetic) and 7 severely frail (completely dependent). It has demonstrated good accuracy, being able to predict death or entry in institutional care of elderly individuals. | Rockwood K, Song X, MacKnight C, et al. A global clinical measure of fitness and frailty in elderly people. Canadian Medical Association Journal 2005; 173(5): 489-95. |
| Smell and Taste Evaluation | The evaluation of integrity of olfactory and gustatory function (according to the patients’ subjective impression) was performed with the aid of Visual Analogue Scale developed by authors, as reported in previous studies. In brief, the patients were asked to indicate their perception of change in the previous ability to recognize (a) smell or (b) taste in a numeric scale ranging from 0 to 10, where higher scores represent better function (0 = unable to identify any (a) smell or (b) taste; 10 = no impairment in (a) smell or (b) taste sensitivity). These scales were administered upon objective, multidisciplinary reassessment of patients 6-11 months after hospital discharge in order to depict patients’ current perception of impairment in smell or taste identification, and also retrospectively to estimate the occurrence of any such impairments during the acute phase of COVID-19. Cut-off scores were used to allocate participants into distinct categories according to magnitude of olfactory and/or gustatory impairment, i.e., severe impairment (0-4); moderate impairment (8-5); mild impairment (9); or no impairment (10) in these chemosensory functions. Subjects presenting with moderate/severe impairment were compared with those reporting mild/no impairment in order to verify the association of these conditions with neuropsychiatric outcomes. Subjects were also inquired about the presence of anosmia, parosmia, cacosmia and fluctuations in smell functions. | McCormack HM, Horne DJ, Sheather S. Clinical applications of visual analogue scales: a critical review. Psychological medicine. 1988;18(4):1007-19. Sayin İ, Yaşar KK, Yazici ZM. Taste and Smell Impairment in COVID-19: An AAO-HNS Anosmia Reporting Tool-Based Comparative Study. Otolaryngol Head Neck Surg. 2020;163(3):473-9. |
| International Physical Activity Questionnaire (IPAQ) – Short Version | Developed by an international board of World Health Organization in 1998, the IPAQ was broadly used and validated in several countries, presenting good psychometric proprieties, including Brazil We used the short version consisting in four questions (with A and B sections) and generated four different levels of physical activity: sedentary, irregularly active, active ad very active. | Craig CL, Marshall AL, Sjöström M, et al. International physical activity questionnaire: 12-country reliability and validity. Med Sci Sports Exerc 2003; 35(8): 1381-95. Matsudo S, Araújo T, Matsudo V, et al. Questionário Internacional de Atividade Física (IPAQ): Estudo de Validade e Reprodutibilidade no Brasil. Rev Bras Ativ Fis Saúde 2001; 6(2). |

**Supplementary Table 3.** Bivariate Analysis between potential predictors and cognitive sub-dimensions with continuous variables.

| **Dependent** | **Independent** | **Correlation** | **Lower CI** | **Upper CI** | **p-value** |
| --- | --- | --- | --- | --- | --- |
| Orientation | Age | -0.235 | -0.302 | -0.165 | < 0.001 |
| Orientation | Charlson Score | -0.220 | -0.289 | -0.149 | < 0.001 |
| Orientation | Basal C-Reactive Protein | -0.034 | -0.109 | 0.042 | 0.386 |
| Orientation | Basal D-Dimer | -0.056 | -0.133 | 0.023 | 0.164 |
| Orientation | Pre-COVID-19 Frailty | -0.303 | -0.368 | -0.235 | < 0.001 |
| Orientation | EGF | -0.004 | -0.107 | 0.100 | 0.946 |
| Orientation | Eotaxin | 0.036 | -0.067 | 0.139 | 0.495 |
| Orientation | G-CSF | 0.054 | -0.049 | 0.156 | 0.306 |
| Orientation | GM-CSF | 0.055 | -0.048 | 0.157 | 0.296 |
| Orientation | IFN-alfa2 | 0.052 | -0.052 | 0.154 | 0.327 |
| Orientation | IFN-gama | 0.056 | -0.048 | 0.158 | 0.291 |
| Orientation | IL10 | -0.018 | -0.121 | 0.085 | 0.728 |
| Orientation | IL12-p40 | 0.049 | -0.055 | 0.151 | 0.356 |
| Orientation | IL12-p70 | -0.031 | -0.133 | 0.073 | 0.561 |
| Orientation | IL13 | -0.010 | -0.113 | 0.093 | 0.844 |
| Orientation | IL15 | 0.064 | -0.040 | 0.166 | 0.227 |
| Orientation | IL17 | 0.047 | -0.056 | 0.150 | 0.369 |
| Orientation | IL1.RA | 0.007 | -0.096 | 0.110 | 0.891 |
| Orientation | IL1-alfa | 0.001 | -0.102 | 0.104 | 0.982 |
| Orientation | IL1-beta | 0.023 | -0.080 | 0.126 | 0.658 |
| Orientation | IL2 | 0.023 | -0.080 | 0.126 | 0.658 |
| Orientation | IL4 | 0.000 | -0.103 | 0.103 | 1.000 |
| Orientation | IL5 | -0.007 | -0.110 | 0.096 | 0.894 |
| Orientation | IL6 | -0.023 | -0.126 | 0.080 | 0.658 |
| Orientation | IL7 | 0.066 | -0.037 | 0.168 | 0.207 |
| Orientation | IL8 | -0.045 | -0.147 | 0.059 | 0.395 |
| Orientation | IP10 | -0.017 | -0.120 | 0.087 | 0.752 |
| Orientation | MCP1 | 0.073 | -0.031 | 0.175 | 0.167 |
| Orientation | MIP1-alfa | -0.216 | -0.312 | -0.115 | < 0.001 |
| Orientation | MIP1-beta | 0.003 | -0.101 | 0.106 | 0.961 |
| Orientation | TNF-alfa | 0.070 | -0.033 | 0.172 | 0.183 |
| Orientation | TNF-beta | 0.001 | -0.102 | 0.104 | 0.987 |
| Orientation | VEGF | 0.082 | -0.021 | 0.184 | 0.117 |
| Orientation | Follow-up C-Reactive Protein | -0.018 | -0.091 | 0.054 | 0.625 |
| Orientation | Follow-up D-Dimer | -0.007 | -0.080 | 0.065 | 0.842 |
| Orientation | Socioeconomic Status (ABEP) | -0.172 | -0.242 | -0.100 | < 0.001 |
| Orientation | Education | 0.235 | 0.114 | 0.350 | < 0.001 |
| Orientation | COVID-19 Severity | -0.036 | -0.108 | 0.037 | 0.275 |
| Orientation | IPAQ | 0.082 | 0.009 | 0.153 | 0.012 |
| Attention | Age | -0.490 | -0.544 | -0.433 | < 0.001 |
| Attention | Charlson Score | -0.438 | -0.497 | -0.376 | < 0.001 |
| Attention | Basal C-Reactive Protein | -0.029 | -0.105 | 0.048 | 0.460 |
| Attention | Basal D-Dimer | -0.126 | -0.203 | -0.047 | 0.002 |
| Attention | Pre-COVID-19 Frailty | -0.302 | -0.368 | -0.233 | < 0.001 |
| Attention | EGF | -0.057 | -0.160 | 0.047 | 0.283 |
| Attention | Eotaxin | -0.068 | -0.171 | 0.037 | 0.203 |
| Attention | G-CSF | 0.148 | 0.045 | 0.248 | 0.005 |
| Attention | GM-CSF | 0.101 | -0.003 | 0.203 | 0.057 |
| Attention | IFN-alfa2 | 0.107 | 0.002 | 0.208 | 0.045 |
| Attention | IFN-gama | 0.087 | -0.017 | 0.189 | 0.102 |
| Attention | IL10 | -0.020 | -0.124 | 0.084 | 0.704 |
| Attention | IL12-p40 | 0.070 | -0.035 | 0.172 | 0.191 |
| Attention | IL12-p70 | 0.019 | -0.085 | 0.123 | 0.720 |
| Attention | IL13 | 0.145 | 0.041 | 0.245 | 0.006 |
| Attention | IL15 | 0.082 | -0.022 | 0.185 | 0.122 |
| Attention | IL17 | 0.019 | -0.086 | 0.123 | 0.725 |
| Attention | IL1-RA | 0.166 | 0.063 | 0.266 | 0.002 |
| Attention | IL1-alfa | 0.133 | 0.029 | 0.233 | 0.012 |
| Attention | IL1-beta | 0.005 | -0.099 | 0.109 | 0.921 |
| Attention | IL2 | 0.042 | -0.062 | 0.145 | 0.431 |
| Attention | IL4 | 0.176 | 0.073 | 0.275 | < 0.001 |
| Attention | IL5 | 0.122 | 0.018 | 0.223 | 0.022 |
| Attention | IL6 | 0.102 | -0.002 | 0.204 | 0.055 |
| Attention | IL7 | 0.196 | 0.093 | 0.294 | < 0.001 |
| Attention | IL8 | 0.021 | -0.083 | 0.125 | 0.690 |
| Attention | IP10 | -0.092 | -0.194 | 0.013 | 0.085 |
| Attention | MCP1 | 0.046 | -0.058 | 0.149 | 0.388 |
| Attention | MIP1-alfa | -0.039 | -0.142 | 0.066 | 0.466 |
| Attention | MIP1-beta | -0.099 | -0.201 | 0.005 | 0.063 |
| Attention | TNF-alfa | 0.087 | -0.018 | 0.189 | 0.103 |
| Attention | TNF-beta | 0.135 | 0.032 | 0.236 | 0.011 |
| Attention | VEGF | 0.154 | 0.051 | 0.254 | 0.004 |
| Attention | Follow-up C-Reactive Protein | -0.150 | -0.221 | -0.077 | < 0.001 |
| Attention | Follow-up D-Dimer | -0.123 | -0.195 | -0.050 | < 0.001 |
| Attention | Socioeconomic Status (ABEP) | -0.299 | -0.365 | -0.230 | < 0.001 |
| Attention | Education | 0.360 | 0.244 | 0.465 | < 0.001 |
| Attention | COVID-19 Severity | -0.045 | -0.118 | 0.029 | 0.123 |
| Attention | IPAQ | 0.121 | 0.048 | 0.193 | < 0.001 |
| Language | Age | -0.420 | -0.478 | -0.358 | < 0.001 |
| Language | Charlson Score | -0.381 | -0.442 | -0.316 | < 0.001 |
| Language | Basal C-Reactive Protein | -0.025 | -0.101 | 0.051 | 0.516 |
| Language | Basal D-Dimer | -0.108 | -0.185 | -0.030 | 0.007 |
| Language | Pre-COVID-19 Frailty | -0.321 | -0.385 | -0.253 | < 0.001 |
| Language | EGF | -0.042 | -0.145 | 0.061 | 0.424 |
| Language | Eotaxin | 0.022 | -0.081 | 0.125 | 0.676 |
| Language | G-CSF | 0.116 | 0.013 | 0.217 | 0.027 |
| Language | GM-CSF | 0.107 | 0.004 | 0.208 | 0.042 |
| Language | IFN-alfa2 | 0.088 | -0.016 | 0.189 | 0.096 |
| Language | IFN-gama | 0.129 | 0.025 | 0.229 | 0.015 |
| Language | IL10 | 0.033 | -0.071 | 0.136 | 0.537 |
| Language | IL12-p40 | 0.088 | -0.016 | 0.190 | 0.096 |
| Language | IL12-p70 | 0.018 | -0.086 | 0.121 | 0.735 |
| Language | IL13 | 0.134 | 0.031 | 0.234 | 0.011 |
| Language | IL15 | 0.136 | 0.033 | 0.236 | 0.010 |
| Language | IL17 | 0.078 | -0.025 | 0.180 | 0.139 |
| Language | IL1-RA | 0.126 | 0.023 | 0.226 | 0.017 |
| Language | IL1-alfa | 0.132 | 0.029 | 0.232 | 0.012 |
| Language | IL1-beta | 0.030 | -0.074 | 0.133 | 0.570 |
| Language | IL2 | 0.076 | -0.027 | 0.178 | 0.149 |
| Language | IL4 | 0.133 | 0.030 | 0.233 | 0.012 |
| Language | IL5 | 0.108 | 0.005 | 0.209 | 0.041 |
| Language | IL6 | 0.082 | -0.022 | 0.183 | 0.123 |
| Language | IL7 | 0.157 | 0.054 | 0.256 | 0.003 |
| Language | IL8 | 0.041 | -0.062 | 0.144 | 0.437 |
| Language | IP10 | -0.045 | -0.148 | 0.059 | 0.394 |
| Language | MCP1 | 0.076 | -0.028 | 0.178 | 0.152 |
| Language | MIP1-alfa | -0.073 | -0.175 | 0.031 | 0.169 |
| Language | MIP1-beta | 0.022 | -0.081 | 0.125 | 0.676 |
| Language | TNF-alfa | 0.090 | -0.013 | 0.192 | 0.088 |
| Language | TNF-beta | 0.131 | 0.028 | 0.231 | 0.013 |
| Language | VEGF | 0.139 | 0.037 | 0.239 | 0.008 |
| Language | Follow-up C-Reactive Protein | -0.080 | -0.152 | -0.008 | 0.030 |
| Language | Follow-up D-Dimer | -0.023 | -0.096 | 0.049 | 0.529 |
| Language | Socioeconomic Status (ABEP) | -0.218 | -0.286 | -0.147 | < 0.001 |
| Language | Education | 0.275 | 0.156 | 0.387 | < 0.001 |
| Language | COVID-19 Severity | -0.025 | -0.098 | 0.047 | 0.380 |
| Language | IPAQ | 0.104 | 0.031 | 0.175 | < 0.001 |
| Epimemory | Age | -0.467 | -0.522 | -0.408 | < 0.001 |
| Epimemory | Charlson Score | -0.416 | -0.475 | -0.352 | < 0.001 |
| Epimemory | Basal C-Reactive Protein | -0.027 | -0.103 | 0.049 | 0.489 |
| Epimemory | Basal D-Dimer | -0.088 | -0.166 | -0.009 | 0.028 |
| Epimemory | Pre-COVID-19 Frailty | -0.259 | -0.326 | -0.188 | < 0.001 |
| Epimemory | EGF | -0.119 | -0.220 | -0.016 | 0.024 |
| Epimemory | Eotaxin | -0.042 | -0.145 | 0.063 | 0.434 |
| Epimemory | G-CSF | 0.129 | 0.025 | 0.229 | 0.015 |
| Epimemory | GM-CSF | 0.021 | -0.083 | 0.125 | 0.690 |
| Epimemory | IFN-alfa2 | 0.091 | -0.013 | 0.193 | 0.085 |
| Epimemory | IFN-gama | 0.095 | -0.009 | 0.197 | 0.072 |
| Epimemory | IL10 | -0.004 | -0.108 | 0.100 | 0.941 |
| Epimemory | IL12-p40 | 0.065 | -0.039 | 0.168 | 0.220 |
| Epimemory | IL12-p70 | -0.018 | -0.122 | 0.086 | 0.735 |
| Epimemory | IL13 | 0.050 | -0.054 | 0.153 | 0.345 |
| Epimemory | IL15 | 0.088 | -0.016 | 0.190 | 0.097 |
| Epimemory | IL17 | 0.014 | -0.090 | 0.117 | 0.795 |
| Epimemory | IL1-RA | 0.115 | 0.011 | 0.216 | 0.030 |
| Epimemory | IL1-alfa | 0.042 | -0.062 | 0.146 | 0.425 |
| Epimemory | IL1-beta | -0.020 | -0.124 | 0.084 | 0.703 |
| Epimemory | IL2 | 0.039 | -0.065 | 0.142 | 0.461 |
| Epimemory | IL4 | 0.065 | -0.039 | 0.168 | 0.218 |
| Epimemory | IL5 | 0.044 | -0.061 | 0.147 | 0.412 |
| Epimemory | IL6 | 0.012 | -0.092 | 0.116 | 0.820 |
| Epimemory | IL7 | 0.132 | 0.029 | 0.233 | 0.012 |
| Epimemory | IL8 | 0.023 | -0.081 | 0.127 | 0.659 |
| Epimemory | IP10 | -0.041 | -0.144 | 0.063 | 0.443 |
| Epimemory | MCP1 | 0.061 | -0.043 | 0.163 | 0.253 |
| Epimemory | MIP1-alfa | -0.150 | -0.250 | -0.047 | 0.005 |
| Epimemory | MIP1-beta | -0.024 | -0.128 | 0.080 | 0.648 |
| Epimemory | TNF-alfa | 0.064 | -0.040 | 0.167 | 0.227 |
| Epimemory | TNF-beta | 0.068 | -0.036 | 0.171 | 0.199 |
| Epimemory | VEGF | 0.146 | 0.042 | 0.246 | 0.006 |
| Epimemory | Follow-up C-Reactive Protein | -0.086 | -0.158 | -0.013 | 0.021 |
| Epimemory | Follow-up D-Dimer | -0.099 | -0.171 | -0.027 | 0.008 |
| Epimemory | Socioeconomic Status (ABEP) | -0.199 | -0.268 | -0.127 | < 0.001 |
| Epimemory | Education | 0.236 | 0.114 | 0.351 | < 0.001 |
| Epimemory | COVID-19 Severity | -0.024 | -0.097 | 0.049 | 0.404 |
| Epimemory | IPAQ | 0.115 | 0.042 | 0.186 | < 0.001 |
| Visuoability | Age | -0.388 | -0.449 | -0.325 | < 0.001 |
| Visuoability | Charlson Score | -0.322 | -0.387 | -0.254 | < 0.001 |
| Visuoability | Basal C-Reactive Protein | -0.002 | -0.078 | 0.074 | 0.965 |
| Visuoability | Basal D-Dimer | -0.112 | -0.190 | -0.034 | 0.005 |
| Visuoability | Pre-COVID-19 Frailty | -0.290 | -0.356 | -0.221 | < 0.001 |
| Visuoability | EGF | -0.097 | -0.199 | 0.007 | 0.067 |
| Visuoability | Eotaxin | -0.048 | -0.151 | 0.056 | 0.364 |
| Visuoability | G-CSF | 0.014 | -0.090 | 0.117 | 0.792 |
| Visuoability | GM-CSF | -0.017 | -0.120 | 0.087 | 0.748 |
| Visuoability | IFN-alfa2 | -0.008 | -0.112 | 0.096 | 0.879 |
| Visuoability | IFN-gama | -0.012 | -0.116 | 0.091 | 0.814 |
| Visuoability | IL10 | -0.056 | -0.159 | 0.048 | 0.290 |
| Visuoability | IL12-p40 | -0.022 | -0.125 | 0.082 | 0.685 |
| Visuoability | IL12-p70 | -0.029 | -0.133 | 0.074 | 0.579 |
| Visuoability | IL13 | 0.055 | -0.049 | 0.158 | 0.296 |
| Visuoability | IL15 | 0.022 | -0.082 | 0.125 | 0.680 |
| Visuoability | IL17 | -0.057 | -0.160 | 0.047 | 0.281 |
| Visuoability | IL1-RA | 0.041 | -0.063 | 0.144 | 0.435 |
| Visuoability | IL1-alfa | 0.041 | -0.062 | 0.145 | 0.434 |
| Visuoability | IL1-beta | -0.041 | -0.144 | 0.063 | 0.437 |
| Visuoability | IL2 | -0.022 | -0.126 | 0.081 | 0.673 |
| Visuoability | IL4 | 0.060 | -0.044 | 0.163 | 0.256 |
| Visuoability | IL5 | 0.054 | -0.050 | 0.157 | 0.309 |
| Visuoability | IL6 | 0.044 | -0.060 | 0.147 | 0.407 |
| Visuoability | IL7 | 0.069 | -0.035 | 0.172 | 0.192 |
| Visuoability | IL8 | 0.060 | -0.044 | 0.162 | 0.261 |
| Visuoability | IP10 | -0.146 | -0.246 | -0.043 | 0.006 |
| Visuoability | MCP1 | 0.054 | -0.050 | 0.157 | 0.307 |
| Visuoability | MIP1-alfa | -0.019 | -0.122 | 0.085 | 0.723 |
| Visuoability | MIP1-beta | 0.030 | -0.074 | 0.133 | 0.577 |
| Visuoability | TNF-alfa | 0.043 | -0.061 | 0.146 | 0.418 |
| Visuoability | TNF-beta | 0.057 | -0.047 | 0.159 | 0.286 |
| Visuoability | VEGF | 0.057 | -0.047 | 0.159 | 0.284 |
| Visuoability | Follow-up C-Reactive Protein | -0.042 | -0.114 | 0.031 | 0.262 |
| Visuoability | Follow-up D-Dimer | -0.021 | -0.094 | 0.052 | 0.570 |
| Visuoability | Socioeconomic Status (ABEP) | -0.230 | -0.299 | -0.159 | < 0.001 |
| Visuoability | Education | 0.270 | 0.150 | 0.382 | < 0.001 |
| Visuoability | COVID-19 Severity | -0.033 | -0.105 | 0.040 | 0.267 |
| Visuoability | IPAQ | 0.081 | 0.008 | 0.153 | 0.005 |
| Cognition | Age | -0.537 | -0.588 | -0.482 | < 0.001 |
| Cognition | Charlson Score | -0.488 | -0.543 | -0.429 | < 0.001 |
| Cognition | Basal C-Reactive Protein | -0.021 | -0.098 | 0.056 | 0.591 |
| Cognition | Basal D-Dimer | -0.133 | -0.211 | -0.054 | 0.001 |
| Cognition | Pre-COVID-19 Frailty | -0.347 | -0.411 | -0.280 | < 0.001 |
| Cognition | EGF | -0.068 | -0.171 | 0.037 | 0.205 |
| Cognition | Eotaxin | -0.037 | -0.142 | 0.068 | 0.486 |
| Cognition | G-CSF | 0.132 | 0.028 | 0.234 | 0.013 |
| Cognition | GM-CSF | 0.086 | -0.019 | 0.190 | 0.106 |
| Cognition | IFN-alfa2 | 0.106 | 0.001 | 0.208 | 0.049 |
| Cognition | IFN-gama | 0.099 | -0.006 | 0.201 | 0.066 |
| Cognition | IL10 | -0.009 | -0.114 | 0.096 | 0.862 |
| Cognition | IL12-p40 | 0.074 | -0.031 | 0.177 | 0.169 |
| Cognition | IL12-p70 | -0.009 | -0.113 | 0.096 | 0.874 |
| Cognition | IL13 | 0.150 | 0.046 | 0.251 | 0.005 |
| Cognition | IL15 | 0.119 | 0.014 | 0.221 | 0.026 |
| Cognition | IL17 | 0.027 | -0.078 | 0.131 | 0.614 |
| Cognition | IL1-RA | 0.157 | 0.052 | 0.257 | 0.003 |
| Cognition | IL1-alfa | 0.138 | 0.034 | 0.239 | 0.010 |
| Cognition | IL1-beta | 0.010 | -0.095 | 0.114 | 0.856 |
| Cognition | IL2 | 0.064 | -0.041 | 0.168 | 0.232 |
| Cognition | IL4 | 0.164 | 0.060 | 0.264 | 0.002 |
| Cognition | IL5 | 0.129 | 0.024 | 0.231 | 0.016 |
| Cognition | IL6 | 0.108 | 0.003 | 0.210 | 0.044 |
| Cognition | IL7 | 0.175 | 0.072 | 0.275 | < 0.001 |
| Cognition | IL8 | 0.049 | -0.056 | 0.153 | 0.358 |
| Cognition | IP10 | -0.102 | -0.205 | 0.003 | 0.057 |
| Cognition | MCP1 | 0.090 | -0.015 | 0.193 | 0.094 |
| Cognition | MIP1-alfa | -0.073 | -0.176 | 0.032 | 0.173 |
| Cognition | MIP1-beta | -0.040 | -0.144 | 0.065 | 0.456 |
| Cognition | TNF-alfa | 0.096 | -0.009 | 0.199 | 0.072 |
| Cognition | TNF-beta | 0.146 | 0.042 | 0.247 | 0.006 |
| Cognition | VEGF | 0.167 | 0.063 | 0.267 | 0.002 |
| Cognition | Follow-up C-Reactive Protein | -0.123 | -0.195 | -0.049 | 0.001 |
| Cognition | Follow-up D-Dimer | -0.086 | -0.158 | -0.012 | 0.023 |
| Cognition | Socioeconomic Status (ABEP) | -0.291 | -0.357 | -0.221 | < 0.001 |
| Cognition | Education | 0.357 | 0.241 | 0.464 | < 0.001 |
| Cognition | COVID-19 Severity | -0.037 | -0.110 | 0.037 | 0.208 |
| Cognition | IPAQ | 0.126 | 0.052 | 0.198 | < 0.001 |

**Supplementary Table 4.** Bivariate Analysis between potential predictors and cognitive sub-dimensions with categorical variables.

| **Dependent** | **Independent** | **Statistic** | **df** | **p-value** |
| --- | --- | --- | --- | --- |
| Orientation | Sex | -2.227 | 694.9 | 0.026 |
| Orientation | Ethnicity | 2.238 | 3, 703 | 0.083 |
| Orientation | Delirium | 1.600 | 340 | 0.111 |
| Orientation | Previous Psychiatric Disease | 1.259 | 509 | 0.209 |
| Attention | Sex | -1.947 | 647.1 | 0.052 |
| Attention | Ethnicity | 2.318 | 3, 684 | 0.074 |
| Attention | Delirium | 1.839 | 331 | 0.067 |
| Attention | Previous Psychiatric Disease | -1.214 | 491 | 0.225 |
| Language | Sex | -2.681 | 726 | 0.008 |
| Language | Ethnicity | 3.883 | 3, 700 | 0.009 |
| Language | Delirium | 2.881 | 339 | 0.004 |
| Language | Previous Psychiatric Disease | -0.412 | 506 | 0.681 |
| Epimemory | Sex | -0.205 | 721 | 0.838 |
| Epimemory | Ethnicity | 1.154 | 3, 695 | 0.327 |
| Epimemory | Delirium | 2.680 | 334 | 0.008 |
| Epimemory | Previous Psychiatric Disease | -0.955 | 501 | 0.340 |
| Visuoability | Sex | -1.396 | 721 | 0.163 |
| Visuoability | Ethnicity | 3.358 | 3, 695 | 0.018 |
| Visuoability | Delirium | 0.978 | 334 | 0.329 |
| Visuoability | Previous Psychiatric Disease | -0.961 | 501 | 0.337 |
| Cognição | Sex | -1.877 | 704 | 0.061 |
| Cognição | Ethnicity | 2.859 | 3, 678 | 0.036 |
| Cognição | Delirium | 2.210 | 327 | 0.028 |
| Cognição | Previous Psychiatric Disease | -0.875 | 485 | 0.382 |

**Supplementary Table 5.** Linear Regression between baseline variables and Global Cognition

|  | **Coefficient** | **SE** | **Lower CI** | **Upper CI** | **p-value** |
| --- | --- | --- | --- | --- | --- |
| (Intercept) | 0.903 | 1.129 | -1.315 | 3.120 | 0.424 |
| Age | -0.069 | 0.010 | -0.090 | -0.049 | < 0.001 |
| Sex [Male] | 0.591 | 0.210 | 0.179 | 1.002 | 0.005 |
| Ethnicity [Yellow] | 1.453 | 1.520 | -1.533 | 4.438 | 0.340 |
| Ethnicity [Black] | 0.120 | 0.375 | -0.617 | 0.857 | 0.749 |
| Ethnicity [Brown] | -0.497 | 0.251 | -0.990 | -0.004 | 0.048 |
| ABEP [B1] | -0.595 | 0.736 | -2.041 | 0.851 | 0.419 |
| ABEP [B2] | -0.234 | 0.687 | -1.583 | 1.115 | 0.734 |
| ABEP [C1] | -0.592 | 0.682 | -1.933 | 0.748 | 0.386 |
| ABEP [C2] | -0.890 | 0.698 | -2.260 | 0.480 | 0.202 |
| ABEP [D-E] | -0.889 | 0.764 | -2.388 | 0.611 | 0.245 |
| Education [Uncompleted Elementary/Middle School] | 2.255 | 0.569 | 1.138 | 3.372 | < 0.001 |
| Education [Completed Elementary/Middle School] | 4.127 | 0.632 | 2.885 | 5.368 | < 0.001 |
| Education [Uncompleted High School] | 4.423 | 0.669 | 3.109 | 5.737 | < 0.001 |
| Education [Completed High School] | 5.231 | 0.607 | 4.038 | 6.423 | < 0.001 |
| Education [Uncompleted Undergraduation] | 6.137 | 0.732 | 4.699 | 7.575 | < 0.001 |
| Education [Completed Undergraduation] | 6.404 | 0.703 | 5.022 | 7.785 | < 0.001 |
| Education [Post-graduation] | 7.481 | 0.820 | 5.870 | 9.092 | < 0.001 |
| Charlson Severity Score | -0.224 | 0.073 | -0.367 | -0.080 | 0.002 |
| Previous psychiatric disease [Yes] | -1.022 | 0.543 | -2.098 | 0.054 | 0.062 |
| Severity WHO [2] | 0.490 | 0.312 | -0.123 | 1.102 | 0.117 |
| Severity WHO [3] | 0.085 | 0.559 | -1.012 | 1.182 | 0.879 |
| Severity WHO [4] | 0.308 | 0.349 | -0.378 | 0.994 | 0.378 |
| Basal C-protein | 0.000 | 0.001 | -0.002 | 0.003 | 0.686 |
| Basal D-dimer | 0.000 | 0.000 | 0.000 | 0.000 | 0.890 |
| Pre-COVID-19 frailty | -0.241 | 0.101 | -0.439 | -0.043 | 0.017 |
| IPAQ [Irregularly Active] | 0.646 | 0.270 | 0.116 | 1.176 | 0.017 |
| IPAQ [Active] | 0.391 | 0.245 | -0.090 | 0.872 | 0.111 |
| IPAQ [Very Active] | 0.703 | 0.538 | -0.352 | 1.759 | 0.191 |

**Supplementary Table 6.** Exploratory Factor Analysis (EFA) with continuous variables from follow up.

| Factor Loadings | | | | | | | | | | | | | | | | | | | | | | | |
| --- | --- | --- | --- | --- | --- | --- | --- | --- | --- | --- | --- | --- | --- | --- | --- | --- | --- | --- | --- | --- | --- | --- | --- |
|  | | | | **Factor** | | | | | | | | | | | | | | |  | | | | |
|  | | | | **1** | | | | **2** | | **3** | | | **4** | | | **5** | | | **Uniqueness** | | | | |
| VEGF | | |  |  | | |  | 0.756 |  |  |  | |  | |  |  |  | 0.3863 | | |  | |  |
| IL7 | | |  |  | | |  | 0.809 |  |  |  | |  | |  |  |  | 0.2658 | | |  | |  |
| IL4 | | |  | 0.879 | | |  |  |  |  |  | |  | |  |  |  | 0.1515 | | |  | |  |
| IFN-alpha2 | | |  | 0.336 | | |  | 0.717 |  |  |  | |  | |  |  |  | 0.3608 | | |  | |  |
| G-CSF | | |  |  | | |  | 0.857 |  |  |  | |  | |  |  |  | 0.1855 | | |  | |  |
| IL5 | | |  | 0.761 | | |  |  |  |  |  | |  | |  |  |  | 0.3357 | | |  | |  |
| IL1-RA | | |  | 0.419 | | |  | 0.774 |  |  |  | |  | |  |  |  | 0.2181 | | |  | |  |
| IL1-alpha | | |  | 0.936 | | |  |  |  |  |  | |  | |  |  |  | 0.0592 | | |  | |  |
| IL13 | | |  | 0.941 | | |  |  |  |  |  | |  | |  |  |  | 0.0454 | | |  | |  |
| IL6 | | |  | 0.930 | | |  |  |  |  |  | |  | |  |  |  | 0.0841 | | |  | |  |
| IL15 | | |  | 0.484 | | |  | 0.564 |  |  |  | |  | |  |  |  | 0.4464 | | |  | |  |
| TNF-beta | | |  | 0.907 | | |  |  |  |  |  | |  | |  |  |  | 0.0934 | | |  | |  |
| Smell | | |  |  | | |  |  |  |  |  | |  | |  | 0.791 |  | 0.3322 | | |  | |  |
| Chronic Fatigue | | |  |  | | |  |  |  | -0.678 |  | |  | |  |  |  | 0.4808 | | |  | |  |
| Taste | | |  |  | | |  |  |  |  |  | |  | |  | 0.750 |  | 0.4003 | | |  | |  |
| C-protein | | |  |  | | |  |  |  |  |  | |  | |  |  |  | 0.9291 | | |  | |  |
| D-Dimer | | |  |  | | |  |  |  |  |  | |  | |  |  |  | 0.9906 | | |  | |  |
| Orientation | | |  |  | | |  |  |  |  |  | | 0.426 | |  |  |  | 0.7996 | | |  | |  |
| Attention | | |  |  | | |  |  |  |  |  | | 0.793 | |  |  |  | 0.3547 | | |  | |  |
| Language | | |  |  | | |  |  |  |  |  | | 0.807 | |  |  |  | 0.3341 | | |  | |  |
| Epi Memory | | |  |  | | |  |  |  |  |  | | 0.616 | |  |  |  | 0.5940 | | |  | |  |
| MCS Patient | | |  |  | | |  |  |  | 0.633 |  | |  | |  |  |  | 0.5623 | | |  | |  |
| FVC | | |  |  | | |  |  |  |  |  | |  | |  |  |  | 0.9543 | | |  | |  |
| Visuoability | | |  |  | | |  |  |  |  |  | | 0.726 | |  |  |  | 0.4668 | | |  | |  |
| Depression | | |  |  | | |  |  |  | 0.823 |  | |  | |  |  |  | 0.2997 | | |  | |  |
| Blood O2 | | |  |  | | |  |  |  |  |  | |  | |  |  |  | 0.9585 | | |  | |  |
| PTSD | | |  |  | | |  |  |  | 0.878 |  | |  | |  |  |  | 0.2153 | | |  | |  |
| Anxiety | | |  |  | | |  |  |  | 0.840 |  | |  | |  |  |  | 0.2875 | | |  | |  |
| *Note.* 'Minimum residual' extraction method was used in combination with a 'varimax' rotation | | | | | | | | | | | | | | | | | | | | | | | |
|  | | | | | | | | | | | | | | | | | | | | | | | |
|  | | | | | | | | | | | | | | | | | | | | | | | |
| **Factor** | | **SS Loadings** | | | | **% of Variance** | | | | | | | | **Cumulative %** | | | | | | | |  |  |
| 1 |  | 5.54 | | |  | 19.77 | | | | | |  | | 19.8 | | | | | |  | |  |  |
| 2 |  | 3.84 | | |  | 13.72 | | | | | |  | | 33.5 | | | | | |  | |  |  |
| 3 |  | 3.16 | | |  | 11.28 | | | | | |  | | 44.8 | | | | | |  | |  |  |
| 4 |  | 2.58 | | |  | 9.22 | | | | | |  | | 54.0 | | | | | |  | |  |  |
| 5 |  | 1.29 | | |  | 4.61 | | | | | |  | | 58.6 | | | | | |  | |  |  |
|  | | | | | | | | | | | | | | | | | | | | | |  |  |
