# Supplementary material for: Cognitive impairment in long-COVID and its association with persistent dysregulation in inflammatory markers
Source: Front Immunol. 2023 May 23;14:1174020. doi: 10.3389/fimmu.2023.1174020 (PMC10242059; doi:10.3389/fimmu.2023.1174020)
Supplement: Supplementary file 2 [file DataSheet_2.docx]

**Supplementary Figure 1.** Confirmatory Factor Analysis (CFA) for Latent Cognitive Dimensions (LCD).
